# Mechanical Performance and Dimensional Stability of Bamboo Fiber-Based Composite

**DOI:** 10.3390/polym13111732

**Published:** 2021-05-25

**Authors:** Yahui Zhang, Wenji Yu, Namhun Kim, Yue Qi

**Affiliations:** 1 Research Institute of Forestry New Technology, Chinese Academy of Forestry, Beijing 100091, China; zhangyhcaf@163.com; 2 Key Lab Wood Science & Technology, State Forestry Administration, Research Institute of Wood Industry, Chinese Academy of Forestry, Beijing 100091, China; chinayuwj@126.com; 3 Department of Forest Biomaterials Engineering, College of Forest and Environmental Sciences, Kangwon National University, Chuncheon 200701, Korea; kimnh@kangwon.ac.kr

**Keywords:** bamboo fiber-based composite, density, dimensional stability, hot press temperature, mechanical properties

## Abstract

The bamboo fiber-based composite (BFBC) has high-performce in terms of mechanical properties and dimensional stability. In this study, BFBCs were prepared with different hot-pressing temperatures (150 °C, 160 °C, 170 °C, 180 °C, 190 °C, and 200 °C) and designed with different densities (1.05 g/cm^3^, 1.10 g/cm^3^, 1.15 g/cm^3^ and 1.20 g/cm^3^), and their selected properties were evaluated. Temperature affected BFBC performance, which, with a general increase in temperature, showed a decrement in mechanical properties and an improvement in dimensional stability. Holocellulose content significantly decreased, and the color of BFBC became darker with the increasing of the press temperature. As the density of BFBC increased, the modulus of elasticity (MOE) significantly increased from 23.09 GPa to 27.01 GPa with the increase in temperature. The thickness swelling ratio (TSR), width swelling ratio (WSR) and water absorption ratio (WAR) declined by more than 30% with the increase in density. Overall, the results of this study provide a theoretical basis and a source of technical support to promote the design, application, and popularization of BFBC in different fields.

## 1. Introduction

Bamboo, as a renewable natural resource, is widely used in many fields because of its fast-growing rate and high strength, and it is a promising material to utilize instead of imported wood. Due to the hollow structure and small section size of bamboo, studies in this area have varied from the use of full culm bamboo to bamboo-based structural products. The utilization of bamboo as a raw material for structural products has been researched, at present, in such applications as laminated bamboo lumber [1], bamboo-oriented strand board [2,3], and bamboo plywood [4,5]. These results showed that the bamboo products have excellent performance including high strength and easy processing, and could be applied as non-structural materials, such as bamboo furniture and indoor flooring, and as heavy load structural materials, such as beams and columns. The degradation and deforestation of forests are a major current issue, and the increasing demand for wood-based panels has caused an important raw material issue in the sector for a long time [6]. Thus, various bamboo products have been studied to improve the utilization of bamboo. Recently, researchers have become interested in a novel bamboo composite (bamboo fiber-based composite) and have carried out investigations of a wide variety of its production processes and properties. Previous studies focused on the significant effects of moisture content on the mechanical properties and dimensional stability of bamboo fiber-reinforced composite [7,8], the effects of thermal treatment on the improvement of the surface properties and chemical properties of bamboo scrimber [9,10,11], the effects of steam treatment of oriented fiber bamboo mat (OBFM) on chemical properties [12], and the effects of different molecular weights of phenol-formaldehyde resin on the infiltration of different bamboo cells of BFRC [13]; staining treatment with acid dye on mechanical and physical properties [14]; hot-and cold-pressing process on properties of BFBC [15], and the basic properties of different bamboo species were also investigated [16].

According to the performance requirements of target products in different fields, designing bamboo fiber-based composite (BFBC) with different properties would be an efficient approach to increase the BFBC application field. The comparison of production parameters for analyzing BFBC could improve the properties and increase the added value of its products. It was indicated that resin content, bamboo species, natural weathering, and thermal treatment can improve the mechanical and dimensional stability of bamboo composite to suit a variety of applications [11,17,18,19]. In the case of production parameters, temperature and density play an important role in the properties of BFBC. However, there is a lack of information that discusses the effects of above two parameters on BFBC in detail. Thus, the objective of this study was to explore the potential suitability of parameters including hot pressing temperature and density of products, and the influence of these two factors on the mechanical properties and dimensional stability of BFBC.

## 2. Materials and Methods

### 2.1. Materials

*Bambusa distegia* (4–6 years old) was obtained from Sichuan, China, and the middle and bottom parts of bamboo were sampled as experimental materials. *Bambusa chungii* McClure bamboo (3 years old) was obtained from Guangdong, China. The phenolic formaldehyde (PF) adhesive was utilized with the following parameters: 47.49% solid content, a viscosity of 37 cps (25 °C), water solubility of 11.2, and a pH of 10.22.

### 2.2. Methods

#### 2.2.1. Manufacturing Processes of Bamboo Fiber-Based Composites (BFBC)

Bamboo raw materials were sawn and split into bamboo-oriented fiber mat (BOFM), then cut with an average length, width, and thickness of 360 mm × 170 mm × 2~5 mm. The obtained *Bambusa distegia* BOFM (BD-BOFM) and *Bambusa chungii* McClure OBFM (BCM-BOFM) were steeped in PF adhesive solution which was diluted to 10% solid content for 3 min, and then dried uniformly to the moisture content of 9%. The resinated BD-BOFM was hot pressed with a pressure of 20 MPa and a rate of 1 mm/min with different temperatures of 150 °C, 160 °C, 170 °C, 180 °C, 190 °C, and 200 °C, and the target density of *Bambusa distegia* BFBC (BD-BFBC) was set as 1.0 g/cm^3^. The glued BCM-BOFMs were hot pressed with a pressure of 20 MPa and the rate of 1 mm/min at 150 °C, and the target densities of *Bambusa chungii McClure* BFBC (BCM-BFBC) were 1.05, 1.10, 1.15, and 1.20 g/cm^3^ (Table 1).

#### 2.2.2. Determination of Mechanical and Physical Properties

The experimental samples were conditioned in a chamber with a temperature of 25 ± 2 °C and a relative humidity of 65 ± 3% for 2 weeks before any measurement was carried out. After conditioning, prepared samples were cut from BFBC and the following mechanical properties were assessed on a universal pressure testing machine (WDW-W10, Time Co. Ltd., Ji Nan, China) and determined in accordance with Chinese Standards: modulus of elasticity (MOE), modulus of rupture (MOR) and shear strength (GB/T 17657) [20]. The compression strength was tested in accordance with ASTM D 3501 [21]. For the evaluation of dimensional stability, the thickness swelling ratio (TSR), width swelling ratio (WSR) and water absorption ratio (WAR) were evaluated according to GB/T 30,364 [22], respectively. The evaluation of TSR, WSR and WAR was conducted using the following equations:

TSR(%) = 100 × (T2 − T1)/T1(1)

where T1 and T2 represent the thickness of the specimens before and after water immersion treatment, respectively.

WSR(%) = 100 × (W2 − W1)/W1(2)

where W1 and W2 represent the width of the specimens before and after water immersion treatment, respectively.

WAR(%) = 100 × (WA2 − WA1)/WA1(3)

where WA1 and WA2 represent the weight of the specimens before and after water immersion testing, respectively.

The entire measurement processes for testing TSR, WSR and WAR were followed as: immersion in boiling water for 4 h, oven-drying at 63 °C for 20 h, and then re-immersion in boiling water 4 h at the final stage. All the measurements were repeated at least five times.

#### 2.2.3. Chemical Analysis

Prior to chemical analyses, samples for each temperature were sawn into small pieces and milled. Fine dust (0.3–0.4 mm) was separated from the samples by sieving. Powder samples were extracted using ethanol-benzene solution; the extractive-free powder samples were delignified using NaClO_2_-acetic acid treatment [23]. The measurement of holocellulose content of BD-BFBCs was calculated using delignified powder samples according to the standard method [24].

#### 2.2.4. Color Change

The color measurement of the samples was performed with a chromameter (CR-400, Konica Minolta Inc., Tokyo, Japan) using an observed angle of 10 degrees and a D64 light source. The sensor head of the spectrophotometer was 8 mm in diameter, with an illuminant C light source and an observed angle of 2°. Each sample, at a different hot press temperature, was measured according to the three parameters of L*, a*, and b*. The total color differences were calculated using the following formula [25],

ΔE* = (ΔL*^2^ + Δa*^2^ + Δb*^2^)^1/2^ (4)

where ΔL*, Δa*, Δb*, and ΔE* represent changes in lightness, red/green chromaticity, yellow/blue chromaticity, and total color change, respectively.

### 2.3. Statistical Analysis

Differences in mechanical and physical properties among samples with different temperatures and densities were statistically examined with a one-way ANOVA and Duncan’s post-hoc tests (IBM SPSS software version 19, SPSS Inc., Chicago, IL, USA).

## 3. Results and Discussion

### 3.1. Mechanical Properties

The results of compressive strength and bending strength of BD-BFBC are displayed in Table 2. The compressive strength slightly decreased from 107.45 to 97.52 MPa with the increasing of the temperature from 150–190 °C. However, the hot press temperature in the range 190–200 °C significantly reduced the compressive strength to 75.31 MPa. The highest reduction of compressive strength was approximately 22.77% when the temperature changed from 190–200 °C, whereas the reduction was less than 5% for every 10 °C decrease from 150–190 °C. The decrement of strength might be mainly attributed to the degradation of chemical components, including the holocellulose and extractive components, which were responsible for the decrease in density, resulting in the decrease in mechanical properties [26]. Consequently, 200 °C was a critical temperature, at which the compressive strength of BFBC was noticeably decreased.

For evaluating the bending strength of BD-BFBC, MOE and MOR values were determined (Table 2). There was no significant difference of MOE between 150 °C and 160 °C, while an obvious decrease was found as temperature increased from 160 to 200 °C. The increasing of temperatures from 150–200 °C had a remarkable effect on MOR of BD-BFBC, with the exception of an increase in temperature from 180–190 °C. The highest reduction in the MOE and MOR was 39.36% and 69.77% as the temperature increased from 190–200 °C, respectively. Compared with the MOE and MOR at 150 °C, the total reduction was 48.06% and 75.67%. Similar results were found in previous studies [27,28], which demonstrated that the MOE and MOR decreased as the treatment temperature increased, and significantly decreased at 200 °C. Moreover, the change of MOE and MOR might have been caused by the alteration in the chemical components. A previous study reported that MOE and MOR were related to the transverse bonding strength among the fibers, which was realized by the connection between cellulose and hemicellulose. As the temperature increased from 150–190 °C, the number of joint points between hemicellulose and cellulose was reduced, which led to the splitting of the intercellular layer, and hence resulted in the decrease in bending strength. Meanwhile, there was an obvious reduction in MOE and MOR as the temperature increased up to 200 °C. It could be mainly explained by the degradation that occurred in hemicellulose, which is less stable at high temperatures than cellulose and lignin [29]. The change in hemicellulose led to additional losses in mechanical properties. According to these results, much higher press temperature is not suitable for manufacturing applications because of a significant reduction in mechanical strength. As similar results showed [29], a reduction in mechanical properties of less than 30% is acceptable for most practical uses, such as decking, or structural applications.

Mechanical properties of BCM-BFBC are also shown in Table 2. The compressive strength of specimens increased from 110.19 to 147.62 MPa with the increasing of the density from 1.05 to 1.20 g/cm^3^. This result could be attributed to increase in the fiber amount or compression ratio, which suggested that the addition of fiber amount might lead to the higher compression ratio. As the compression ratio increased, the bonding point would be increased to join the adhesive with bamboo fiber [15]. A significant increase was observed in MOE as the density of composites increased. The range of data in the MOE was from 23.09 to 27.01 GPa. With regard to the MOR, density had a negative effect on those values. The MOR value increased significantly from 176.50 MPa to 199.93 MPa with the change in density from 1.05 to 1.10 g/cm^3^, but was not noticeable for the increase from 1.10 g/cm^3^ to 1.20 g/cm^3^. The remarkable improvement of MOE and MOR could be generalized as follows: the load-bearing properties of bamboo mainly depend on the bamboo fiber. Due to the densification of bamboo cell tissues, the fiber content per unit of volume increases. On the other hand, after the mechanical fluffing process, the bamboo veneer becomes thinner and there are many linear shaped cracks which increase the contact area with adhesive [30]. Once the bamboo thin-walled cells were broken with the fluffing machine, the redistribution of internal stress occurred, which rapidly transferred the load on the broken cells to the surrounding tissues. In general, the mechanical properties of composites mainly depended on the structure and strength of cellulosic fibers, which were increased through structural densification, resulting in high endurance capacity for these composites. Consequently, appropriate press temperature and density should be considered for the improvement of mechanical properties.

### 3.2. Dimensional Stability

**Figure 1:**
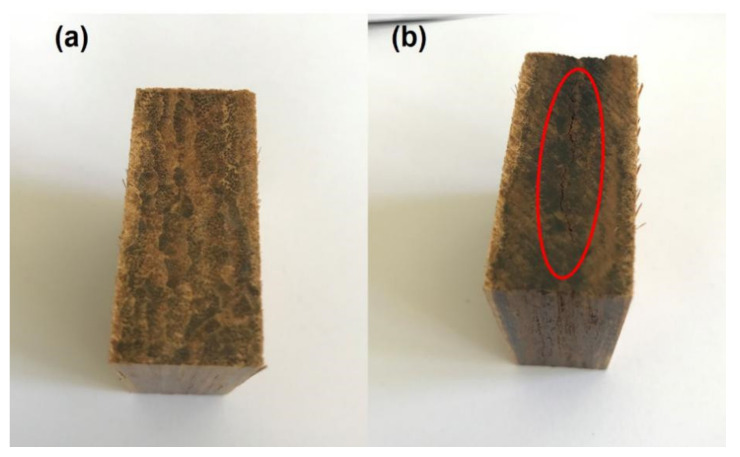
(**a**): Sample produced at a hot press temperature of 180 °C without crack; (**b**): obvious crack in sample produced at a hot press temperature of 200 °C.

**Figure 2:**
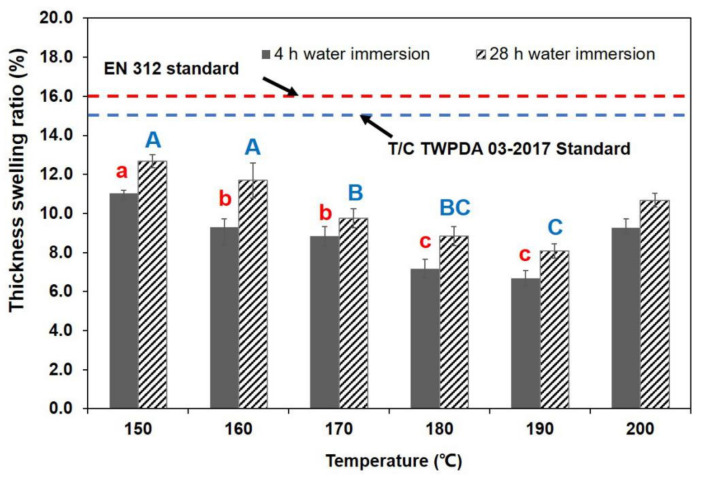
Thickness swelling results at different hot press temperatures. (Notes: The same lowercase (4 h water immersion) and capital letter (28 h water immersion) are not significantly different at 5% significance level using Duncan’s post-hoc test).

**Figure 3:**
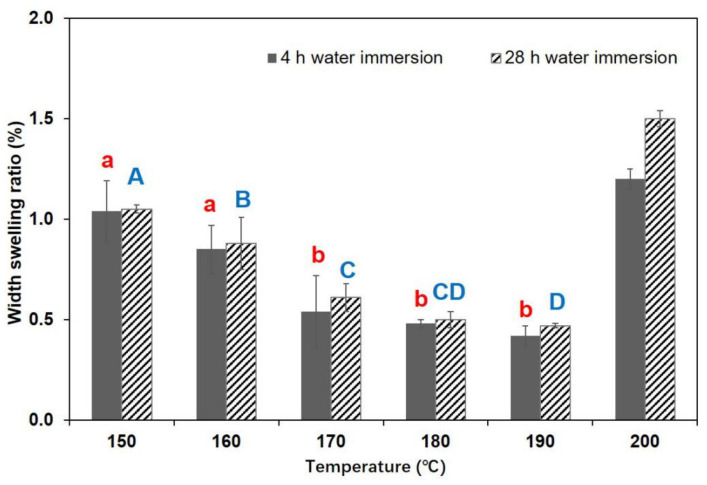
Width swelling results at different hot press temperatures (Notes: The same lowercase (4 h water immersion) and capital letter (28 h water immersion) are not significantly different at 5% significance level using Duncan’s post-hoc test).

**Figure 4:**
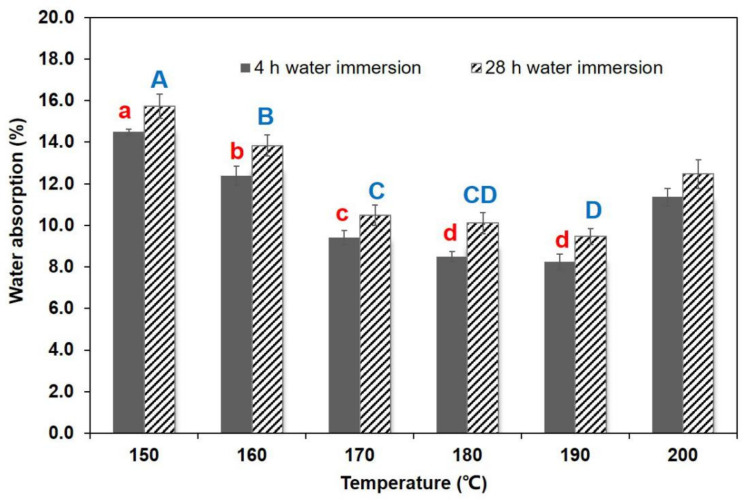
Water absorption results at different hot press temperatures (Notes: The same lowercase (4 h water immersion) and capital letter (28 h water immersion) are not significantly different at 5% significance level using Duncan’s post-hoc test).

The increase in TSR and WAR values was attributable to the carbonization reaction in the core layer, which caused the cracks to appear in the specimen (Figure 1). Figure 2 and Figure 3 display the TSR and WSR values of BD-BFBC at different hot press temperatures. Both the TSR and WSR of the samples measured in the 4 h water immersion test were mostly lower than those in the 28 h water immersion test. The springback of the BFBC, as they were soaked in water, was transferred in less-dimensional stability, which is a common behavior of any wood composite [31]. For all experimental samples, TSR is much higher than WSR due to the high levels of compression in the direction of thickness of the bamboo. In comparison with width direction, the thickness direction was more prone to recovery after hot pressing. Besides, the TSR and WSR values, in the 4 h test, were influenced by temperature, which shows a declining trend with increasing temperature in the range of 150–190 °C. The TSR and WSR decreased from 11.04% to 6.67% and from 1.04% to 0.47% in the 4 h immersion test, and ranged from 12.68% to 8.09% and 1.05% to 0.48% in the 28 h immersion test, respectively. In case of WAR values, they decreased significantly from 14.49% to 8.24% in the 4 h immersion test, and from 15.73% to 9.46% for the 28 h test, respectively (Figure 4). The improvement in dimensional stability could be mainly attributed to the alteration of hemicellulose, which is the most heat sensitive polymer. As the press temperature increased, the destruction of hemicelluloses caused a decrease in the hygroscopicity of the BFBCs [32,33]. These change of hemicelluloses with increasing press temperature resulted in the decrease in hygroscopicity of the bamboo materials, specifically the enhancement of the water resistance. However, TSR and WSR values suddenly increased as the press temperature increased from 190–200 °C, and were still lower than those at a press temperature of 150 °C. The cracks can easily lead to water soaking. Compared to the standard of oriented strand board [34] and particle board [35], the maximum TSR values of BD-BFBC at 28 h were lower than those of load-bearing boards, the values of which were 16% and 15% in the 24 h immersion test, respectively.

**Figure 5:**
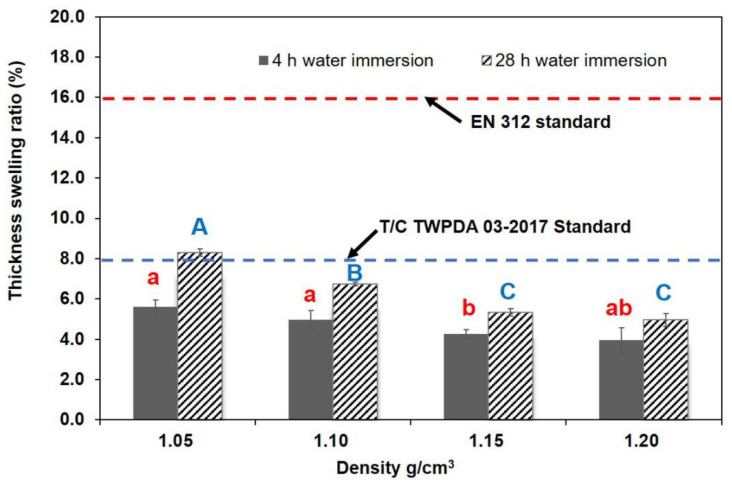
Thickness swelling results at different hot press temperatures (Notes: The same lowercase (4 h water immersion) and capital letter (28 h water immersion) are not significantly different at 5% significance level using Duncan’s post-hoc test).

**Figure 6:**
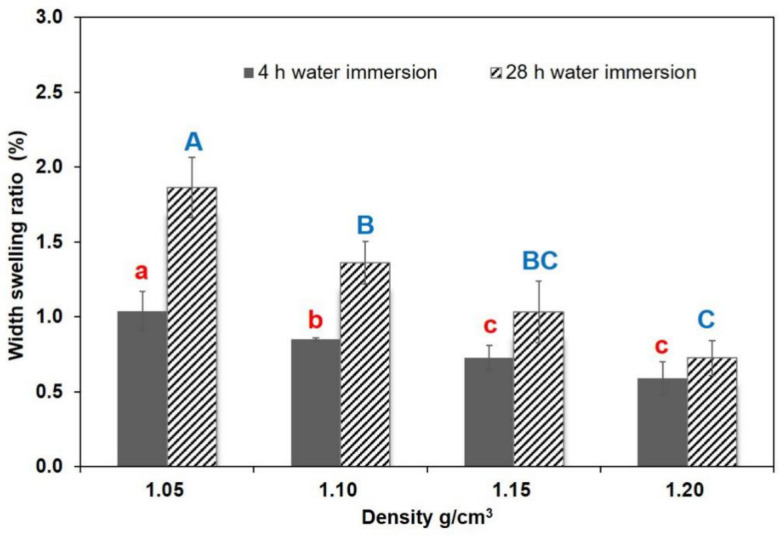
Width swelling results at different hot press temperatures (Notes: The same lowercase (4 h water immersion) and capital letter (28 h water immersion) are not significantly different at 5% significance level using Duncan’s post-hoc test).

**Figure 7:**
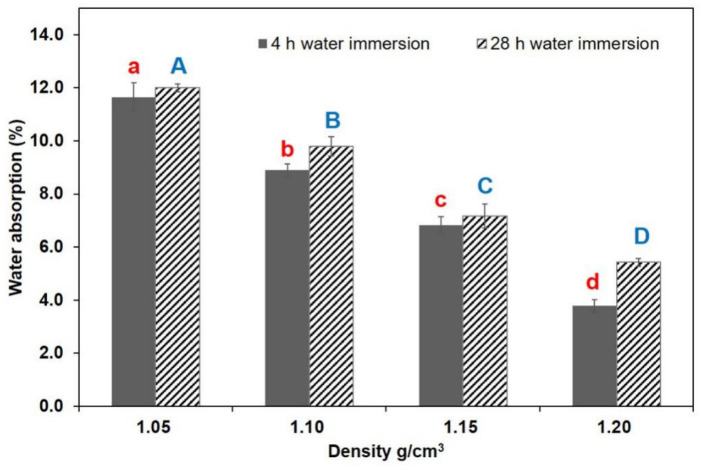
Water absorption results at different hot press temperatures (Notes: The same lowercase (4 h water immersion) and capital letter (28 h water immersion) are not significantly different at 5% significance level using Duncan’s post-hoc test).

Figure 5, Figure 6 and Figure 7 shows water resistance after 4 h and 28 h soaking in water. The correlation between composite density and water resistance was analyzed. TSR showed no significant decrease in samples with increasing densities in the 4 h immersion test, but it decreased considerably when the density increased from 1.05 to 1.15 g/cm^3^ in the 28 h test. WSR values showed a declining trend as density increased to 1.15 g/cm^3^ in the 4 h test, and obvious difference in WSR was noticeable as density increased from 1.05 to 1.20 g/cm^3^ in the 28 h test. In the case of the 4 h water immersion test, the decrements of TSR and WSR values ranged from 5.60% to 3.96%, and 1.04% to 0.59%, respectively. Compared to the 4 h test, the results of the TSR and WSR values in the 28 h water immersion test were, on average, higher, showing decrements from 8.30% to 4.96%, and 1.87% to 0.73%, respectively. Significant differences were observed in WAR values between the specimens that underwent an increase in density, with a range of 11.65% to 3.78%, and 12.00% to 5.43%, in the 4 h and 28 h water tests, respectively. The improvement in the WAR of specimens at elevated temperatures could be due to the decreased porosity in the composite. The higher density of some samples could lead to a reduction in the likelihood of material to absorb water, and a change in its path, which could increase the bonding strength between the resin and the material. In addition, the increase in density effectively reduced the accumulation of internal stress during densification and prevented the compressed deformation cells from springing back due to internal stress release under the high moisture condition. Based on EN standards, particleboard should have a maximum TSR value of 8% for 2 h immersion and 15% for 24 h immersion (EN312-4), respectively. The average TSR values of BCM-BFBC specimens were 5.60% for 4 h immersion and 8.30% for 28 h immersion, which were much lower than the above values. Furthermore, the TSR and WSR of BCM-BFBC samples reached the GB/T 30364-2013 standard for bamboo scrimber products (TSR ≤ 10%, WSR ≤ 4%). Overall, the increase in press temperature and density has a positive effect on the enhancement of dimensional stability, but the appropriate temperature and density should be selected according to the demand of the actual manufacturing equipment used and the economic cost this entails.

### 3.3. Changes in Chemical Composition

The chemical composition of BCM-BFBC differed from 150–200 °C (Table 3). A decrease in the holocellulose content with increasing temperature was found. The holocellulose content ranged from 57.41% to 52.43%, respectively. The decrease in holocellulose content is due to an effect caused by the reduction in polysaccharide content. Because of its branched structure and amorphous tissues, polyoses are considerably more susceptible to thermal degradation than wood components [36]. Regarding hemicellulose, the content decreased significantly from 16.52% to 10.26%. It has been proven that hemicelluloses are less resistant to thermal degradation than cellulose and lignin because of their low molecular weight and branching structures at high temperature [37]. In addition, high temperature induces a decrease in arabinose, galactose, xylose, and mannose contents, which mainly originated from the degradation of carbohydrates, which are products of the decrement of hemicellulose [38,39]. According to the results of this study, the decrease in hemicellulose caused the alteration of mechanical properties and dimensional stability, which significantly reduced bending strength and hydroscopic properties.

### 3.4. Color Changes

**Figure 8:**
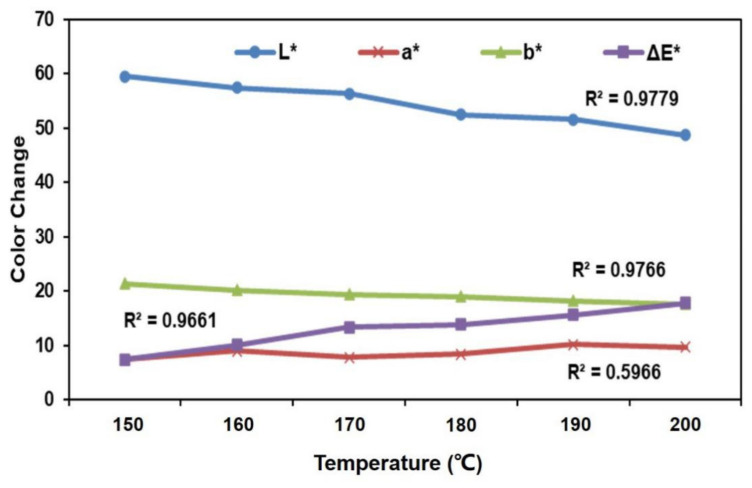
Color parameters of BFBC manufactured at different temperatures.

Color change is one of the most important aesthetic aspects of natural biomaterial. The color differs not only between various species, but also within one species. The color changes in BCM-BFBC, at different press temperatures, are displayed in Figure 8. Some color parameters exhibited a linear relationship with temperature. As the temperature increased, the lightness (L*) and the yellow-blue chromaticity (b*) decreased. The correlation coefficients (R^2^) of these parameters were 0.9779 and 0.9766, respectively. However, the red-green chromaticity (a*) was not significantly affected by the changing temperature. ΔE* increased with an increase in hot press temperature and was highly influenced by the lightness behavior [40,41]. It was established that the changes in color appeared mostly due to a reduction in lightness, which was related to the degradation of hemicelluloses (Table 2).

Moreover, it was also proposed that ΔE* results from chemical changes in lignin due to the darkening of lignin, which was associated with the generation of chromophoric groups, and mainly with the increase in carbonyl groups. Additionally, the changes in color with the increasing of the temperature could be attributed to the oxidation of phenolic compounds, the presence of reduced sugars and amino acids, the emanation of formaldehydes, the formation of quinines, or the caramelization of holocellulose components [42,43].

## 4. Conclusions

Hot press temperature and density were the main parameters that influenced the properties of BFBC. In general, the bending properties of BD-BCM decreased with the increasing of the hot press temperature. This is mainly due to the degradation of the hemicellulose. However, the results showed that the dimensional stability of BD-BFBC was improved by elevated temperatures, which, in particular, significantly influenced the water absorption. As the temperature increased up to 200 °C, an obvious reduction in mechanical properties was found due to the mass loss of chemical components. In addition, a slight increase in swelling and water absorption was observed owing to the occurrence of cracks in BD-BFBC at the highest temperature. For the color parameters of BD-BFBC, the L* and b* values decreased with the increase in temperature, and the ΔE* value showed a converse trend that affected by changes in temperature. However, hot press parameters had no impact on the a* value. As density increased, the compressive strength and MOE of BCM-BFBC showed an increasing trend. The BCM-BFBC with 1.20 g/cm^3^ showed the best water resistance. With regard to the obtained results, it could be calculated that optimum temperature and density are very important in the control of mechanical properties and dimensional stability, and also enhance the serviceability of products.

## Figures and Tables

**Table 1:** Details on experiments.

Raw Materials	Variables of Production	Test Schematic and Setup
*Bambusa distegia*	Temperature (150 °C, 160 °C, 170 °C, 180 °C, 190 °C, 200 °C)	Bending strength
		Comprehensive strength
		Water resistance
		Chemical components
		Color change
*Bambusa chungii McClure*	Density (1.05 g/cm^3^, 1.10 g/cm^3^, 1.15 g/cm^3^, 1.20 g/cm^3^)	Bending strength
		Comprehensive strength
		Water resistance

**Table 2:** Mechanical properties of BD-BFBC produced at different hot press temperatures.

Specimens	Parameters	Compressive Strength (MPa)	MOE (GPa)	MOR(MPa)
	150 °C	107.45(1.19) ^A^	21.35(0.54) ^A^	207.19(2.07) ^A^
	160 °C	105.90(2.94) ^A^	21.01(0.46) ^A^	198.70(21.03) ^AB^
BD-BFBC	170 °C	101.06(2.53) ^AB^	19.59(0.90) ^B^	180.77(11.07) ^BC^
	180 °C	99.14(2.27) ^B^	19.00(0.97) ^BC^	177.01(6.90) ^C^
	190 °C	97.52(0.96) ^B^	18.32(0.10) ^C^	170.05(4.85) ^C^
	200 °C	75.31(2.72) ^C^	11.09(0.41) ^D^	51.63(2.13) ^D^
	1.05 g/cm^3^	110.19(1.09) ^A^	23.09(1.54) ^A^	176.50(13.89) ^A^
BCM-BFBC	1.10 g/cm^3^	116.12(3.74) ^A^	24.28(1.60) ^AB^	199.93(4.81) ^B^
	1.15 g/cm^3^	137.10(5.62) ^B^	25.56(0.66) ^B^	201.34(14.62) ^B^
	1.20 g/cm^3^	147.62(1.78) ^C^	27.01(0.72) ^C^	210.86(16.07) ^B^

Notes: Means within a column followed by the same capital letter are not significantly different at 5% significance level using Duncan’s post-hoc test. Numbers in parenthesis are standard deviations.

**Table 3:** The holocellulose content at different hot press temperature conditions.

Temperature	Holocellulose (%)	Hemicellulose (%)
150 °C	57.41(0.01) ^A^	16.52(0.15) ^A^
160 °C	55.75(0.21) ^B^	15.45(0.21) ^B^
170 °C	55.73(0.13) ^B^	15.04(0.03) ^C^
180 °C	54.97(0.02) ^C^	13.15(0.09) ^D^
190 °C	53.62(0.05) ^D^	12.63(0.10) ^E^
200 °C	52.43(0.01) ^E^	10.26(0.11) ^F^

Notes: Means within a column followed by the same capital letter are not significantly different at 5% significance level using Duncan’s post-hoc test. Numbers in parenthesis are standard deviations.

## Data Availability

Not applicable.
